# Caribbean climate change vulnerability: Lessons from an aggregate index approach

**DOI:** 10.1371/journal.pone.0219250

**Published:** 2019-07-10

**Authors:** Roxann K. Stennett-Brown, Tannecia S. Stephenson, Michael A. Taylor

**Affiliations:** Department of Physics, The University of the West Indies, Mona, Jamaica, West Indies; Columbia University, UNITED STATES

## Abstract

The study examines the potential influence of sub-regional variations in climate, and specifically heavy rain events, in determining relative vulnerabilities of locations in twelve Caribbean countries. An aggregate vulnerability index, referred to as the Caribbean Vulnerability Score (CVS), is created using historical demographic and socioeconomic data and climate data representing extreme rain events. Four scenarios are explored. Firstly, comparative vulnerabilities are determined when heavy rainfall is incorporated in CVS versus when it is excluded. The impact of climate change is also investigated using future climate data derived from statistical downscaling but holding demographic and socioeconomic sub-indices constant. The analysis is repeated with projections of future demographic structure from the Shared Socioeconomic Pathway data (SSP3), future climate projections and constant socioeconomic. Finally, the sensitivity of the results is examined with respect to applying different weights i.e. versus using equal weights for the climate and non-climatic components of CVS as is done for the first three scenarios. Results suggest that the inclusion of historical susceptibility to rainfall extremes influences relative vulnerabilities within the Caribbean when compared to the rankings of vulnerability derived using only socioeconomic and demographic inputs. In some cases significant increases in relative rankings are noted. Projected changes in the intensity of rain events across the Caribbean region in the 2030s and 2050s, do not significantly alter the top and lowest ranked vulnerable locations when demographic and socioeconomic indices are held constant. Changes may however occur in the order of the top ranked locations dependent on scenario and time slice. In general, future shifts in relative vulnerabilities were found to be dependent on (i) changes in both future climate and demographic scenarios, (ii) the time horizons being considered, and (iii) the weighting assigned to climate in the future.

## 1. Introduction

Small island developing states (SIDS), including those in the Caribbean, are among the most vulnerable to climate change [1]. A growing body of research suggests that for the region as a whole, climate changes already evident include more intense rain events, longer dry spells, higher and more frequent extreme temperatures and rising sea levels (see for e.g. [1–3]). Other studies suggest a general intensification of these changes in the future under increased global warming projections (see for e.g. [4–7]). Vulnerability is, however, not uniformly distributed among the countries of the Caribbean, [8] due to variations in different factors governing exposure and resilience e.g. geography, demographics, economic ability to withstand and cope with disasters, and exposure to extreme climatic events. This paper attempts to explore the role of intra-regional variation in exposure to climate extremes when determining comparative vulnerabilities within the Caribbean.

Heavy rain events which occur over relatively short periods (sometimes associated with hurricanes and tropical storms but many times not), are a particular challenge for Caribbean territories. Events can occur yearly and repeatedly in a single year with far reaching impact (see for example Table 1 for Jamaica). To explore how exposure to heavy rainfall extremes may influence comparative vulnerabilities within the Caribbean we define a simple index of vulnerability premised firstly on sub-indices representing socioeconomic well-being and demographic structure for 12 countries (see Section 2). The impact made on the relative rankings of vulnerability when climate sub-indices representing heavy rainfall events are also included in the simple index is then examined. (This analysis is hereafter referred to as Experiment 1). The index comprising the socio-economic, demographic and climate sub-indices is called the Caribbean Vulnerability Score (CVS). The formulation of CVS is further discussed in Section 2 (Methodology) and its potential significance as an indicator of vulnerability is discussed in Section 5.

**Table 1:** Some extreme rain events impacting Jamaica and their relative costs.

Event	Year	Category	Cost (J$ billions)	Impact (%GDP)
Hurricane Michelle	2001	4	2.52	0.8
May/June Flood Rains	2002		2.47	0.7
Hurricane Charley	2004	4	0.44	0.02
Hurricane Ivan	2004	3	36.9	8.0
Hurricane Dennis and Emily	2005	4	5.98	1.2
Hurricane Wilma	2005	5	3.6	0.7
Hurricane Dean	2007	4	23.8	3.4
Tropical Storm Gustav	2008		15.5	2.0
Tropical Storm Nicole	2010		20.6	1.9
Hurricane Sandy	2012	1	9.7	0.8
March-June Rains	2017		4.0	0.2

Source: Planning Institute of Jamaica.

**Fig 1:**
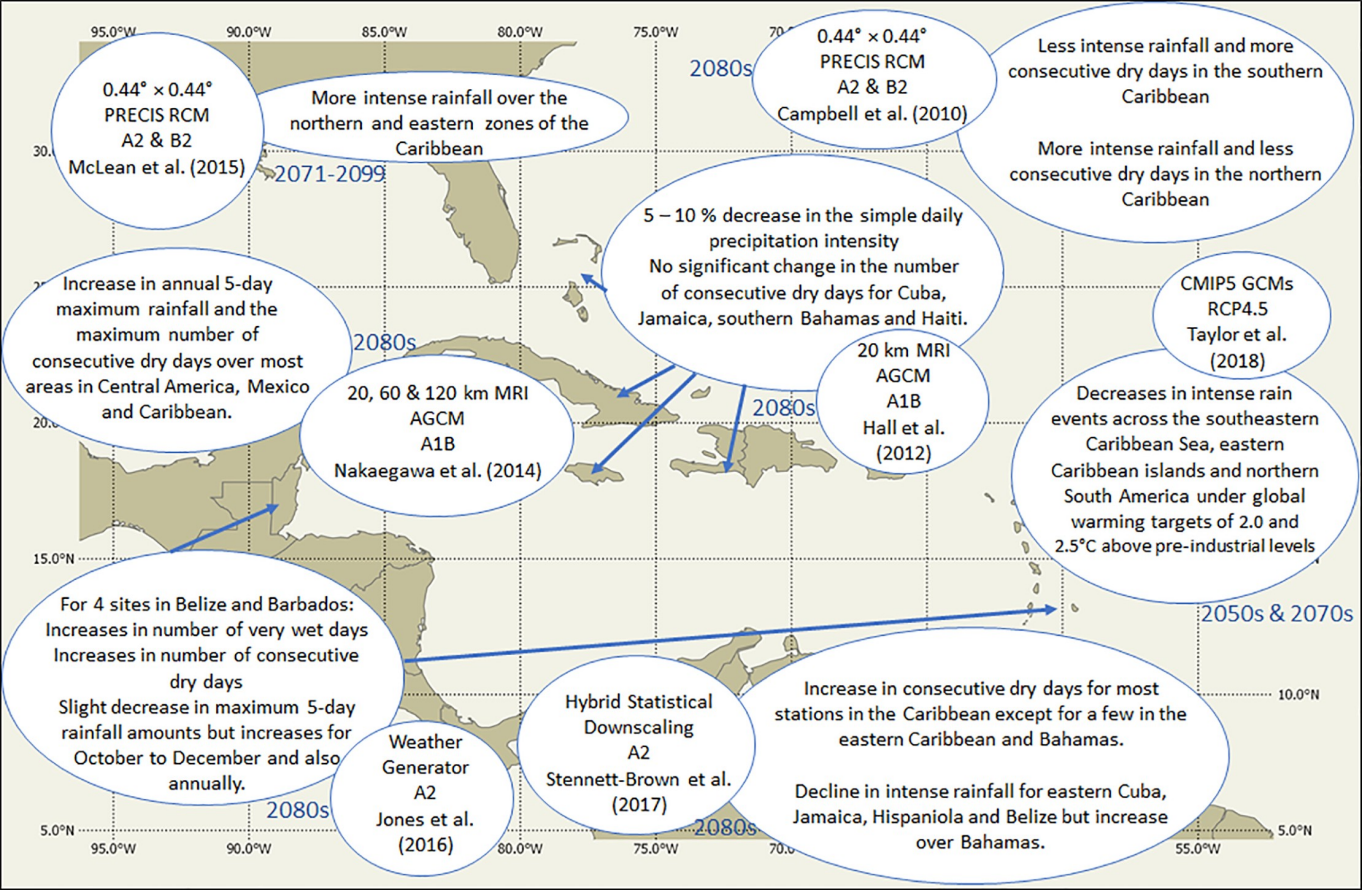
Summary of projections of rainfall extremes for the Caribbean.

Global warming is also projected to significantly alter climate in the Caribbean region. End of century projections include increases in annual land and ocean surface temperatures of 1.0 to 3.5°C; changes in annual rainfall ranging between approximately −50 and +13.7% and mean sea level rise of up to 1.4m [4,9–12]. Projected changes in daily rainfall extremes from a number of different studies are summarized in Fig 1. Most of the studies use various downscaling mechanisms to better capture intra-regional variations in climate (i.e. as opposed to using global circulation models), including a high resolution global circulation model [11], regional climate models [4, 13], a weather generator [14] and statistical models from a hybrid of stochastic and regression approaches [15]. The results suggest some intra-regional variation in sign and magnitude of change, including:

- more intense rainfall and less consecutive dry days over the northern Caribbean with the opposite pattern over the south under the A2 scenario for 2071–2100 [4];
- a tendency towards more intense rainfall for northern and eastern Caribbean countries across the A2 and B2 emission scenarios for 2071–2099 with exceptions over zone 1 (northern Cuba and Bahamas) under the A2, zone 4 (eastern Caribbean) under the B2 and zone 5 (Trinidad and Guyana) under the A2 and B2, and with even less consensus on length of dry and wet spells [13].
- an increase in annual maximum 5 day rainfall and consecutive dry days over most areas in Central America, Mexico and Caribbean under the A1B scenario for 2075–2099 [16];
- an increase in the number of very wet days and a slight decrease in maximum 5 day rainfall for Belize for 2041–2070 and Barbados for 2011–2040 under the A1B [14];
- an increase in consecutive dry days for most Caribbean stations except over some eastern Caribbean locations and Bahamas and decreases in annual maximum 1 day rainfall, annual count of days with daily rainfall above 10 mm and annual total rainfall above the 95^th^ percentile over some northern stations and Belize, with increases and decreases over the eastern Caribbean under the A2 scenario for 2071–2099 [15];
- increases and decreases in the simple daily rainfall intensity dependent on sub-region [11] under A1B for 2075–2099;

In this study statistical downscaling is used to determine changes in the heavy rainfall-sub-indices used in CVS for defined future periods (see Section 2). The climate projections are used to investigate the sensitivity of the relative vulnerabilities determined by CVS to global warming induced changes in the heavy rainfall profile of the Caribbean. To the best of the authors’ knowledge this is the only study to date that attempts to quantitatively incorporate climate model projected change in an aggregate vulnerability index for this region. The investigation is first done using the future climate projections with demographic and socio-economic sub-indices held constant (Experiment 2). The analysis using future climate is then repeated for two other scenarios–firstly with future demographic projections also incorporated and socioeconomic data held constant (Experiment 3); and secondly with different weightings applied to the climate (changing) versus non-climatic (constant) sub-indices (Experiment 4). The weightings used in Experiment 4 are explained in Section 2. All prior experiments assumed equal weightings for the sub-indices used in CVS. The aims of Experiments 2, 3 and 4 are respectively to examine (i) if projected changes in intra-regional variations in heavy rainfall due to global warming may alter present-day relative vulnerability rankings; (ii) the comparative influence of future changes in both climatic and non-climatic factors (relative to the present-day); and (iii) the potential sensitivity of the relative vulnerability assessments to the amount of influence assigned to the climate (changing) versus non-climatic (constant) sub-indices when formulating CVS.

The paper is organized as follows: Section 2 presents the methods and data used including the formulation of the CVS. Section 3 explores results of Experiment 1 (historical analyses). Section 4 discusses projections for climate as deduced by statistical downscaling and projections of future demography. Section 5 presents the results from Experiments 2 and 3 (changing climate only and changing climate and non-climate inputs). The results of Experiment 4 (equal versus unequal weightings and future climate) are also presented in Section 5. Section 6 discusses some implications and limitations of the results in the broader context of the use of aggregate indices as a means of characterising vulnerability within an already highly vulnerable region.

## 2. Methodology and data

### 2.1 Experiment 1: Historical vulnerability

**Fig 2:**
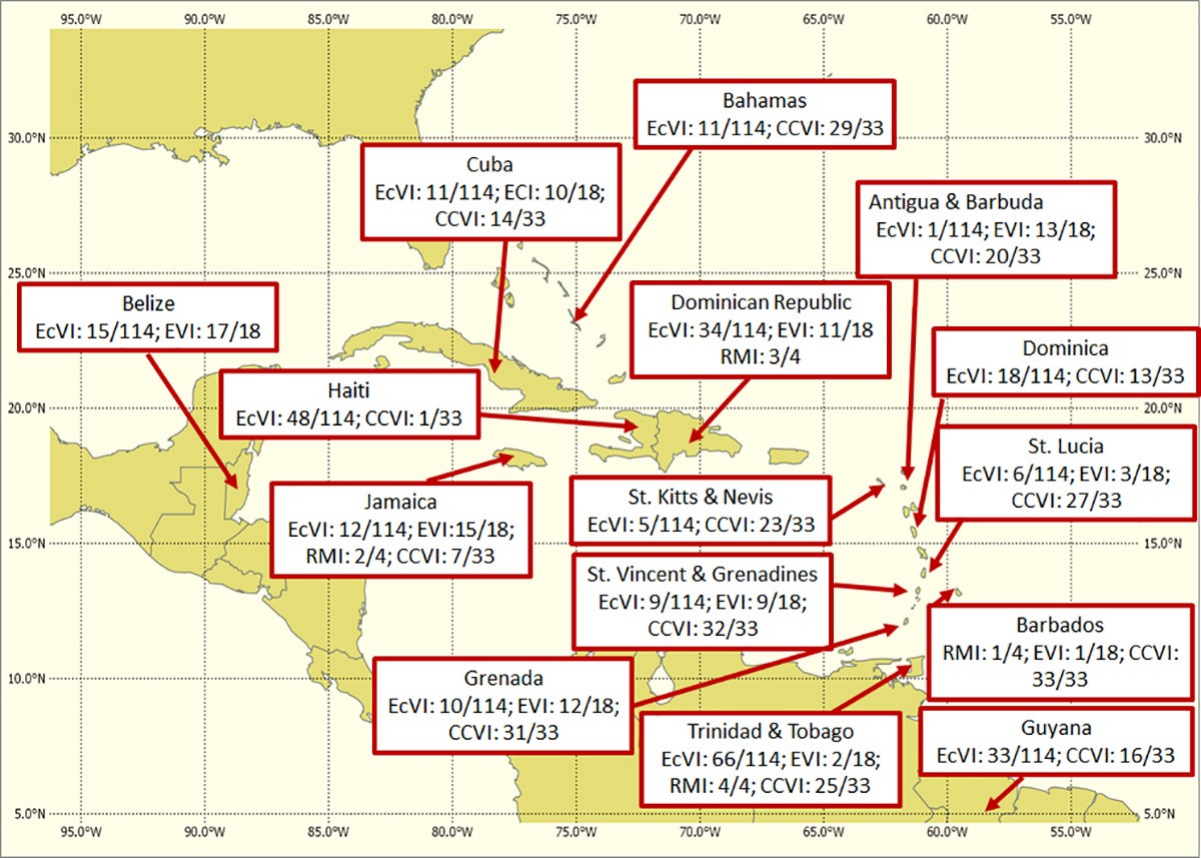
Vulnerability rank of some Caribbean countries. EcVI is the economic vulnerability index [17]. EVI is the Environmental Vulnerability Index [18]. RMI is the Risk Management Index [19] and CCVI is the Climate Change Vulnerability Index [20]. A value of 1/114 indicates the highest vulnerability of the 114 countries in the study.

In this study vulnerability is considered the degree to which a system is susceptible to, or unable to cope with adverse effects of climate change, including climate variability and extremes. A few scientific articles and many reports from financial and regional institutions document the vulnerability of SIDS with respect to climate variability or change at a national level. Some of these investigations are summarized in Table 2 and Fig 2 and provide broad guidance for the Caribbean vulnerability index developed for this study.

**Table 2:** A subset of vulnerability indices applied at a national scale for SIDs that incorporate natural disasters, climate variability or climate change.

Reference	Index	Motivation	Sub-indices	Weighting
[20]	Climate Change Vulnerability Index (CCVI)	To provide insights into the vulnerability of countries by quantifying potential impacts of climate change and climate-related extremes	1. Exposure; 2. Sensitivity; 3. Adaptive capacity	Indicator 1–50% Indicator 2–25% Indicator 3–25%
[21]	Risk Management Index (RMI)	To provide a way to identify risk management capacities, as well as comparative data for evaluating the effects of policies and investments on risk management	1. Risk identification; 2. Risk reduction; 3. Disaster management; 4. Governability and financial protection	Equal
[21]	Disaster Deficit Index (DDI)	To provide a measure of country risk from a macro-economic and financial perspective when faced with possible catastrophic events	1. Volume and cost of exposed elements to disasters (Maximum Considered Event); 2. Stock of available funds for recovery (Economic Resilience)	Equal
[22]	Economic Vulnerability Index (EVI-G)	To provide an assessment of the vulnerability of the SIDs or to identify the least developed countries (LDCs)	3 shock sub-indices e.g. instability of goods and services and instability of agriculture production due to natural disaster; 4 exposure sub-indices e.g. smallness of population size, remoteness	Shock indicators– 50% Exposure indicators– 50%
[18]	Environmental Vulnerability Index	To reflect the extent to which the natural environment of a country is prone to damage and degradation	50 sub-indices e.g. wind, dry, wet, slides, relief, low lands, air, waste, fisheries	Equal
[23]	Commonwealth Vulnerability Index (CVI)	To provide a tool to complement additional criterion (e.g. per capita income) for determining whether small states should be accorded differential treatment by the international community.	1. Lack of diversification; 2. Export dependence; 3. Impact of natural disasters; 4. Resilience	Two elements comprise index: vulnerability index and resilience index. Indicators1-3 are weighted using principal component analysis (PC) and combined with the resilience PCA.
[17]	Economic Vulnerability Index EcVI	To examine the exposure of an economy to exogenous shocks arising out of its inherent characteristics e.g. smallness	1. Exposure to foreign economic conditions; 2. Remoteness and insularity; 3. Disaster proneness	Two sets: 1. Equal 2. Indicator 1–50% Indicator 2–40% Indicator 3–10%

The studies all utilize aggregated vulnerability indices/scores that incorporate a range of indicators including proneness to natural disasters [17], risk of exposure to climate change and extreme events e.g. [20, 18] and economic losses that can be incurred with catastrophic events e.g. [19]. An extensive review of country and community level vulnerability work for SIDS and the Least Developed Countries (LDC) is provided by [24]. Of note, [20] is the only report examined that includes climate change projections for the Latin America and the Caribbean in its aggregate approach. The report, however, does not focus on an increase or decrease to baseline climate parameters but rather measures the degree of change as representative of the necessity for that system (human or natural) to cope with a potential alteration of the current state.

The Caribbean vulnerability measure, CVS, used in this study, is formulated using the aggregate approach. CVS is first formulated using two sub-indices: demographic structure and socio-economic wellbeing. Each sub-index is obtained by doing a simple arithmetic average across its respective normalized indicators. There are three indicators for demographic structure (population density, dependent youth population, aged population) and four for socioeconomic wellbeing (gross domestic product growth rate, purchasing power parity, education, longevity). The demography and socioeconomic datasets for 2006–2011 are obtained from World Bank (2016) from http://databank.worldbank.org/data/home.aspx. Table 3 presents the sub-indices, their component indictors and hypotheses guiding their uses. Each indicator value is obtained by averaging the annual data series over the period 2006–2011.

**Table 3:** Summary of sub-indices and their indicators used in the compilation of the Caribbean Vulnerability Score (CVS).

Sub-index	Component indicators	What each indicator represents	Hypothesized functional relationship between the indicator and vulnerability	Data source
Exposure to intense rain events	Maximum 1-day rainfall	Annual highest daily precipitation (in mm)	The greater the annual highest daily rainfall, the greater the vulnerability	[3]
	Maximum 5-day rainfall	Annual highest 5 consecutive days precipitation (in mm)	The greater the annual highest 5 consecutive days rainfall, the greater the vulnerability	[3]
	Days above 10 mm	Annual count of days when rainfall is greater than 10 mm	The greater the annual count of days when rainfall is greater than 10 mm, the greater the vulnerability	[3]
Demographic Structure	Population density	Measure of population per unit area (km^2^)	The higher the population density, the greater the vulnerability	World Bank (2016)
	Dependent youth population	Percent population 0–14 years of age	The higher the dependent population, the greater the vulnerability	World Bank (2016)
	Aged population	Percent population ≥65 years of age	The higher the aged population, the greater the vulnerability	World Bank (2016)
Economic wellbeing and stability	Gross Domestic Product (GDP) Growth (Annual %)	A proxy for the vitality of an economy	The higher the GDP growth rate the less the vulnerability	World Bank (2016)
	Purchasing power parity	Exchange rate conversion factor which takes into account price differences between countries	The higher the purchasing power parity, the less the vulnerability	World Bank (2016)
	Education	Gross enrolment ratio, secondary, both sexes	The higher the gross enrolment ratio, the less the vulnerability	World Bank (2016)
	Longevity	Life expectancy at birth (in years)	The higher the longevity, the greater the vulnerability	World Bank (2016)

The indicator value for a country is normalized relative to the values of the same indicator for other countries. This allows the combination of different indicators in the relevant sub-index on a common scale for a given country. The countries used in this study are Antigua, Bahamas, Barbados, Belize, Cuba, Dominican Republic, Grenada, Guyana, Jamaica, St. Lucia, St. Vincent and Trinidad & Tobago. The sampling of countries is largely guided by the availability of demographic, socioeconomic and climate data, but is also meant to capture a wide range of relative vulnerabilities (see again Fig 1 for the vulnerability ranks for the countries for some indices described in Table 1). In this study, normalized values for a specific indicator are obtained by subtracting its minimum value across all 12 countries from each country’s indicator value then dividing by the difference between the maximum and minimum values identified across all 12. See Eq (1). A similar normalization approach is proposed by [25].

$$X_{j}=\frac{X_{i}-X_{min}}{X_{max}-X_{min}}$$ (1)

where X_j_ = normalized country indicator

X_i_ = country indicator where i denotes the country

X_min_ = minimum value of the indicator across the region

X_max_ = maximum value of the indicator across the region

The normalized data have a range of 0 to 1 with 1 representing the highest level of vulnerability. In cases where after normalization the sub-index shows an inverse association i.e. 1 representing the lowest level of vulnerability, the data are transformed by subtracting the normalized sub-index from 1. The socioeconomic sub-indices such as education, longevity and GDP were transformed. The CVS is then formulated using equal weightings for the two sub-indices.

In the second formulation of CVS, a climate sub-index is included. The three indicators comprising the sub-index are maximum 1 day rainfall, maximum 5 day rainfall and days above 10 mm. Historical daily rainfall data are sourced from a meteorological station in each of the 12 countries. It is important to note that only one station per country is used in this study. Whereas for some smaller islands the station may be representative of general conditions across the entire country, it is recognized that for the larger territories it is likely not. We discuss this limitation further in the discussion section but note here that (i) data limitations restrict creating country-scale indices for all the considered territories at this time, and (ii) the aim is to show the relative influence of including sub-regional variation in climate indices, which is still facilitated using the station data.

**Fig 3:**
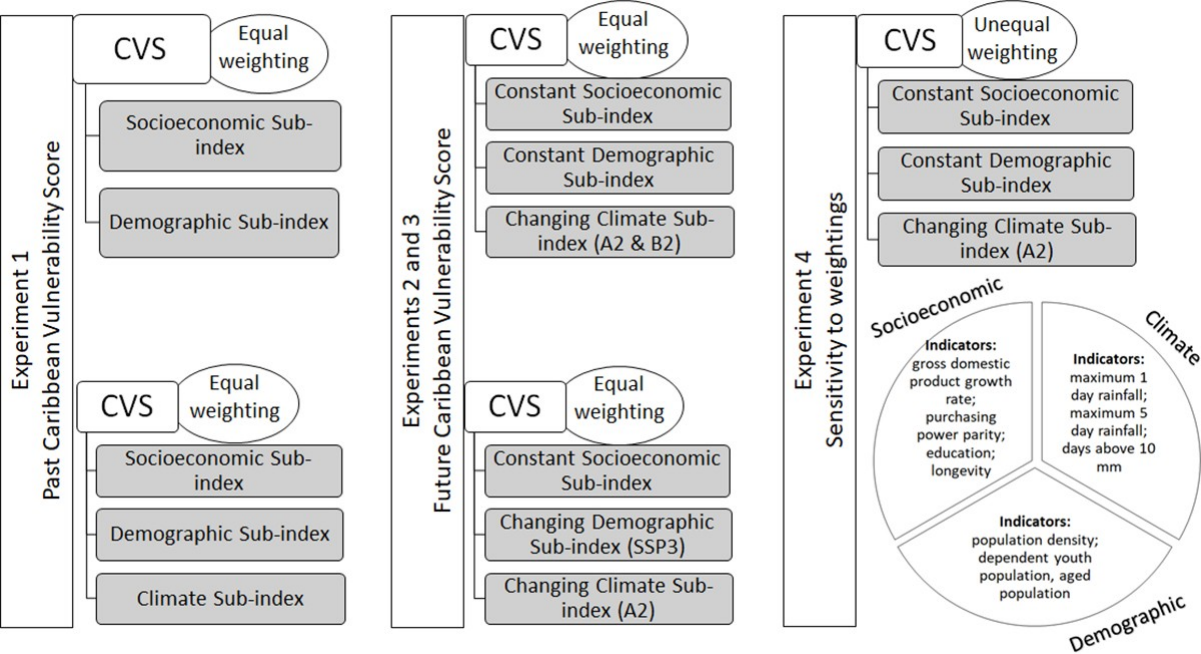
Schematic on approaches used in study.

The daily data quality and homogeneity were assessed previously by [3]. Data for the period 1986–2010 are used for the calculation of three annual extreme rainfall timeseries using the RClimDex software. RClimDex is a user friendly graphical interface that facilitates the computation of up to 27 core ETCCDI (Expert Team on Climate Change Detection and Indices) extreme indices [26]. The indicator value for each country is obtained by averaging values of extremes timeseries over 2006–2011. The 2006–2011 period is the span over which demographic, socioeconomic and climate data are available across all 12 countries. As before the sub-index is calculated using an arithmetic average over the climate indicators. Fig 3 shows a schematic of the approach.

### 2.2 Experiments 2 and 3: Future vulnerability

Near term (2030s) and medium term (2050s) intense rain sub-indices are created for each station. The rain sub-index for each future period and for each location is created by calculating the arithmetic mean of the normalized future extreme rain indicators i.e. RX1, RX5 and R10. To determine future values of a rain indicator, a change factor is added to the historical indicator value (originally averaged over 2006–2011). Change factors are obtained from analyses of daily rainfall projected for 1961–2099 under the A2 and B2 emissions scenarios for each station using Statistical Downscaling Model (SDSM) [27].

SDSM uses a hybrid statistical downscaling approach that incorporates multiple linear regression and weather generator schemes to create statistical models between predictors (e.g. geopotential height, relative humidity) and local station variables (rainfall). The statistical models are used to produce future daily rainfall time series. Statistical models for rainfall are created and validated using predictors from NCEP/NCAR Reanalysis [28] for 1961–2001. The models are then used to generate future scenarios of daily rainfall using predictors from the Hadley Centre Coupled Model version 3 (HadCM3) [29] global climate model outputs for 1961–2099 under the A2 and B2. Predictors are provided for a gridbox over or in closest proximity to the respective stations. [15] provide further details on model creation and validation and an analysis of their skills by zones across the Caribbean. The annual extreme rainfall indices are calculated from the model daily rainfall output for 2001–2015 (baseline), 2030–2039 (near term) and 2050–2059 (medium term) using RClimDex.

The change factor is obtained by calculating the difference between the average annual indicator value for the model baseline period and the average for each future period. The change factor is then applied to the historical intense rain indicator to obtain the future indicator value. For each location the future values of RX1, RX5 and R10 are then normalized (relative to other station values for the specific indicator) and combined to create the intense rain sub-index. In Experiment 2, this future sub-index is aggregated with the historical demography and socio-economic sub-indices to explore future comparative vulnerabilities across the region due only to a change in climate conditions. This process is undertaken for the SRES A2 (medium high emissions) and B2 (medium low emissions scenarios).

In Experiment 3, future vulnerability scores are obtained by considering both future climate and future demographic data with the socioeconomic indicator held constant. The future demographic data are obtained online through the publicly available interactive Shared Socioeconomic Pathway (SSP) web-database at https://tntcat.iiasa.ac.at/SspDb. The SSP3 was selected because of its equivalence to the SRES A2. SSP3 describes a world dominated by regional rivalry and shares many scenario characteristics with the fragmented world of SRES A2 [30]. Data are available for 8 of the 12 countries—Bahamas, Belize, Barbados, Cuba, Dominican Republic, Guyana, Jamaica, Trinidad and Tobago. The data are obtained for the years 2030 and 2050. The database provided data for the population but not the population density hence the population density was calculated by dividing the population total by the country size in square kilometres. The future demographic indicators are normalized and combined with the normalized future climate and constant socioeconomic sub-index to produce future vulnerability scores for the 8 countries.

### 2.3 Experiment 4: Sensitivity of weighting of sub-indices

The final analyses examine the sensitivity of the results to the weightings used. This is examined for the changing climate under the A2 scenario for the mid-century with constant demographic and socioeconomic data. The weightings of 25%, 40% and 35% are assigned to exposure to intense rain events, demographic structure and socio-economic wellbeing respectively. The results are then compared with results from Experiment 2 which is identically premised but with equal weightings. The weightings are premised on the study of [31] who suggest similar percentages for climate versus non-climate exposure and resilience sub-indices, based on literature review and a series of discussions with experts actively involved in developing useful adaptation and mitigation strategies for addressing climate change.

## 3. Results

### 3.1 Experiment 1

**Fig 4:**
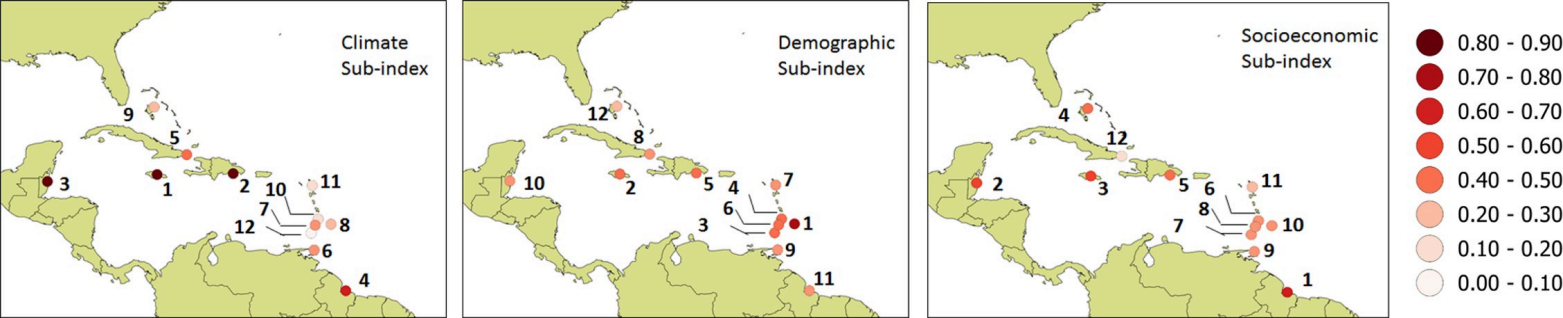
Spatial distribution of ranking of countries based on each sub-index.

Fig 4 shows the ranking of the 12 locations examined for each of the three sub-indices separately. There is considerable variation in relative ranking dependent on the sub-index used. For the climate sub-index the most vulnerable locations in order of decreasing vulnerability are located in Jamaica, Belize and Dominican Republic. For the demographic data, the most vulnerable locations in the study are in Barbados, Jamaica and Grenada while for socioeconomic input Guyana, Belize and Jamaica are identified as the most vulnerable. The least vulnerable locations are in Bahamas, Cuba and Grenada for demographic, socioeconomic and climate indicators respectively.

**Fig 5:**
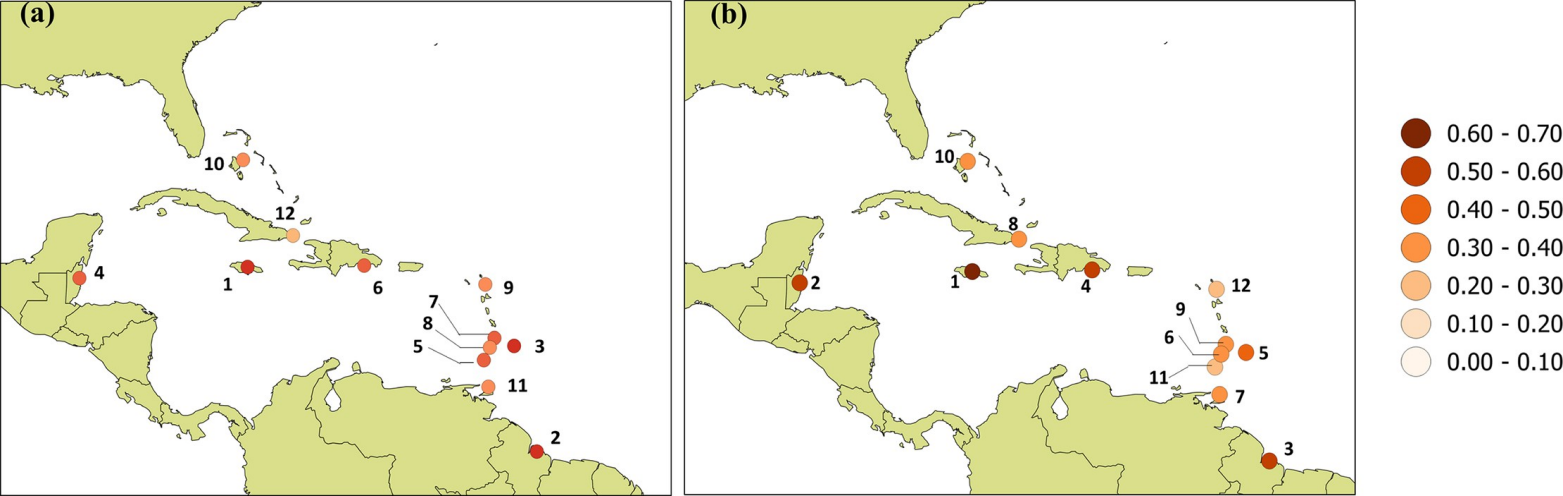
Ranking of stations using CVS. Calculated from historical data (2006–2011) using (a) demographic and socioeconomic only and (b) demographic, socioeconomic and climate.

When vulnerability is examined as an aggregate function of demographic and socioeconomic inputs only, Jamaica, Guyana and Barbados are identified as the most vulnerable with Cuba as the least vulnerable (Fig 5A). When intense rain events are factored in, Jamaica retains its rank as the most vulnerable, but is followed by Belize and Guyana, while Antigua and Barbuda emerge as the least vulnerable (Fig 5B). In fact, all locations, with the exceptions of Jamaica (1^st^) and Bahamas (10^th^) have a shift in their suggested comparative vulnerability. Locations whose vulnerabilities increase when heavy rains are considered are Belize (4^th^ to 2^nd^), Dominican Republic (6^th^ to 4^th^), St. Vincent (8^th^ to 6^th^), Trinidad (11^th^ to 7^th^) and Cuba (12^th^ to 8^th^). The initial suggestion is that the inclusion of a sub-index which captures intra-regional climate variability, (as represented by intense rains in this case), changes the comparative vulnerability of Caribbean countries to climate in comparison to rankings based on demographic and socioeconomic conditions alone.

### 3.2 Experiments 2 and 3

#### 3.2.1 Validation of climate indicators from statistical models

**Fig 6:**
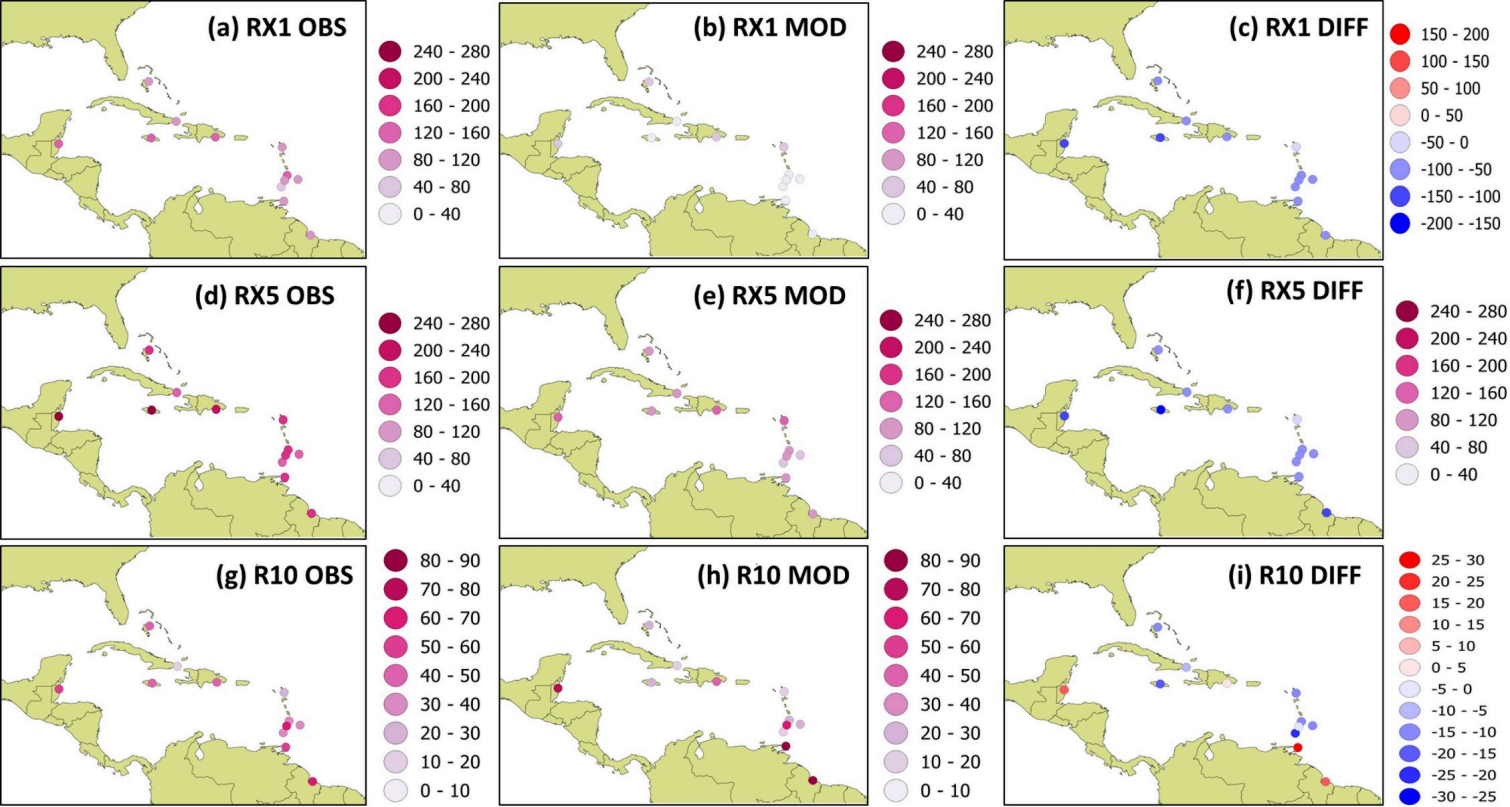
Annual extreme rainfall averages calculated over 1985 to 2001 for station observations and statistical models. Panels (a)-(c) are for RX1; Panels (d)-(e) are for RX5; Panels (g)-(i) are for R10. Left panel shows station observations; middle panel statistical models and right panel shows models minus observations.

Future vulnerability is initially examined using rainfall projections from statistical models created in SDSM. Table 4 shows the model predictors of daily rainfall for each station. The models’ outputs and their derived extreme indices are validated as detailed in [15]. Biases of the average annual values of the maximum 1 day rainfall (RX1), 5 day rainfall (RX5) and days above 10 mm (R10) relative to station observations for the 12 stations used in this study are shown in Fig 6. The models underestimate the extreme indices by up to approximately 110 mm and 163 mm for RX1 and RX5 respectively. R10 is overestimated over Dominican Republic (1 day), Belize (16 days), Guyana (19 days) and Trinidad (26 days) and underestimated over the other Caribbean locations by up to 22 days. Positive and significant correlations between the modelled versus observed values of R10 are obtained for most stations ranging from 0.28 to 0.71 (See S1 Appendix). Exceptions are St. Lucia (-0.14), St. Vincent (-0.04) and Trinidad (0.02). Significant and positive correlations are obtained for RX1 and RX5 only for Jamaica, Cuba, Antigua and Dominican Republic i.e. largely over northern locations. In general, though biases exist in representing the extreme indicators, there is reasonable representation of the average annual variability by the statistical models for some locations, particularly those in the northern Caribbean.

**Table 4:** Model predictors retained for rainfall models created in SDSM for 12 stations used in the vulnerability study.

Country	Station	Model Predictors
Antigua	VC Bird	Surface vorticity, 850 hPa meridional velocity, Relative humidity at 500 hPa
Bahamas	Nassau	Surface vorticity, Surface Specific Humidity, Relative humidity at 500 hPa
Barbados	CIMH	Surface zonal velocity, Surface vorticity, Relative humidity at 500 hPa
Belize	PSWGIA	850 hPa airflow strength, 850 hPa divergence, Relative humidity at 500 hPa, 850 hPa vorticity
Cuba	Maisi	500 hPa vorticity, Relative humidity at 500 hPa, Relative humidity at 850 hPa, Surface divergence
Dominican Republic	Santo Domingo	Surface vorticity, Relative humidity at 500 hPa, 850 hPa vorticity, 850 hPa zonal velocity
Grenada	MBIA	Surface specific humidity, Surface vorticity, Relative humidity at 500 hPa
Guyana	Georgetown	Relative humidity at 500 hPa, 850 hPa zonal velocity, Surface divergence
Jamaica	Worthy Park	Relative humidity at 500 hPa, 850 hPa vorticity, 850 hPa zonal velocity
St. Lucia	Hewanorra	Surface vorticity, 850 hPa airflow strength, Surface specific humidity, Relative humidity at 500 hPa
St. Vincent	Joshua	Surface vorticity, Relative humidity at 500 hPa, 850 hPa meridional velocity
Trinidad and Tobago	Piarco	Surface vorticity, Surface specific humidity, Relative humidity at 500 hPa

#### 3.2.2 Projected change in climate indicators

**Fig 7:**
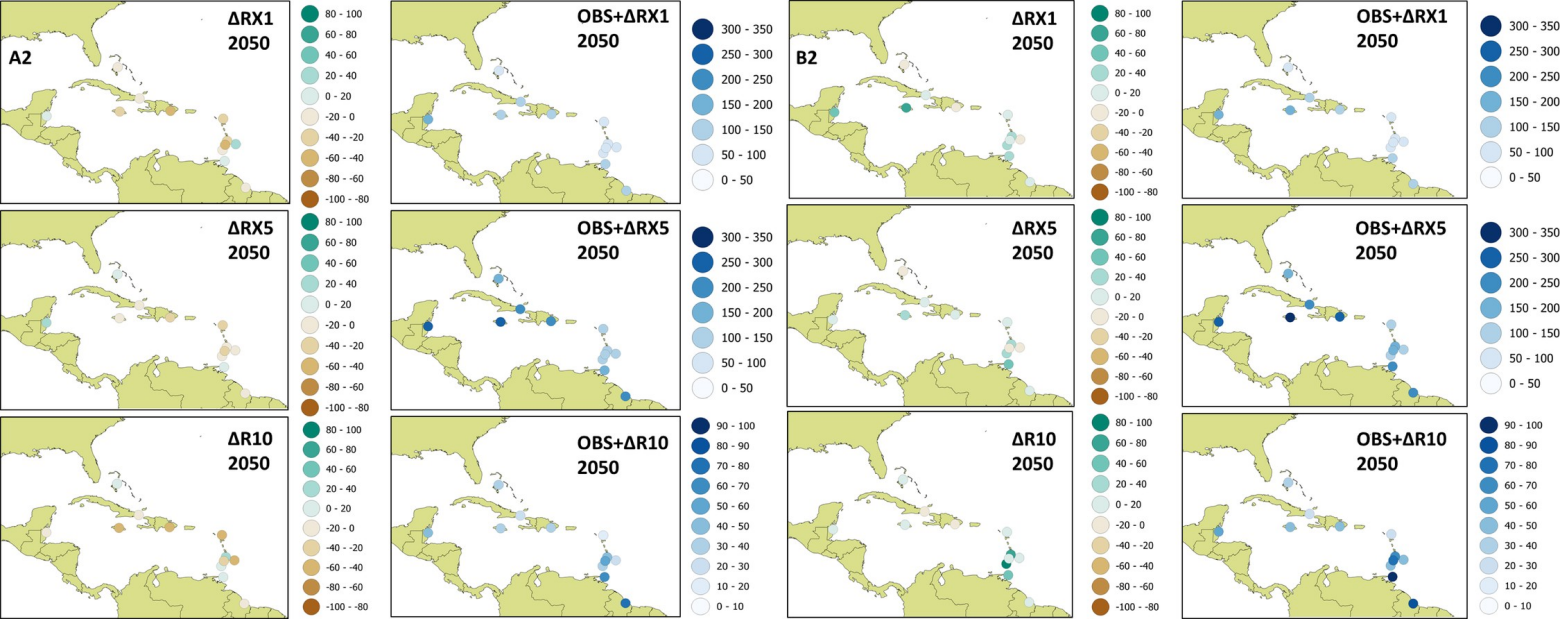
Future scenarios of RX1, RX5 and R10 for the 2050s under the A2 and B2 scenarios.

Projections for the near term (2030s) under the A2 scenario suggest decreases in maximum 1-day rainfall (RX1), maximum 5-day rainfall (RX5) and days above 10 mm (R10) for most of the 12 locations (See S2 Appendix). Strongest decreases are indicated for some eastern Caribbean countries for RX1 and RX5 with values of up to 47% (St. Vincent) and 30% (Antigua) respectively. The greatest decrease in R10 is noted for the station in Jamaica at approximately 43%. The only stations for which increases are suggested are in Trinidad (for RX1), St. Lucia (for R10) and Bahamas (for RX5 and R10). For the medium term (2050s), decreases in RX1, RX5 and R10 continue to be the dominant response under the A2 (Fig 7). Positive changes are, however, suggested for a few locations including Trinidad and Belize for all three indices and for Bahamas, Barbados, Grenada and St. Lucia for at least one of the indices.

Under the lower emissions scenario B2, the suggestion is generally that of increases in the rainfall intensities over most stations. Increases are up to 17% (RX1) and 22% (RX5) as observed for Trinidad for 2030s and up to 42% (RX1) and 43% (RX5) as observed for Belize and Trinidad respectively for 2050s. Days above 10 mm are generally expected to decrease by up to 24 days (Barbados) for the 2030s but largely shift towards an increase by up to 51 days (Jamaica) by the 2050s.

#### 3.2.3 Projected change in demographic indicators

SSP3 projections suggest an increase in total population, and by extension, the population density for all countries except Cuba and Barbados. A decrease in population density is projected for Cuba to the end of century relative to 2010. Projections for Barbados suggest a peak in population density by 2030 and subsequent declines through to the end of century. Four of the eight countries show an increase in the dependent youth populations. These countries were Bahamas, Belize, Jamaica and Dominican Republic. The greatest increase is projected for Dominican Republic and greatest decrease is for Cuba. All 8 countries show an increase in the aged population with the largest change projected for Cuba by 2030 and 2050 relative to 2010.

#### 3.2.4 Future Caribbean vulnerability scores: Changing climate only

The normalized future climate sub-index values are obtained from future RX1, RX5 and R10 values (i.e. observed values + change factors) under the A2 and B2 scenarios for the 2030s and 2050s. Values for the 2050s are shown in Fig 7. The future sub-index values provide some initial insights into comparative vulnerabilities across the 12 countries with respect to future intense rain events only (i.e. not considering demographics or socio-economic sub-indices). Under the A2 scenario, locations most vulnerable to intense rains are Jamaica, Guyana and Belize for the 2030s in order of decreasing vulnerability. However by the 2050s, though the locations in Belize and Guyana are still identified as the most vulnerable, only Guyana retains its rank. The location in Trinidad is now identified as the 3^rd^ most vulnerable of the 12 countries. In fact the relative vulnerabilities suggested for 8 of the locations change between the 2030s and 2050s and only the locations in the Guyana (2^nd^), Cuba (6^th^), Bahamas (7^th^) and St. Lucia (10^th^) retain their relative rank. Under the B2 scenario, the climate sub-index deduced for the 2030s suggest that Belize, Dominican Republic and Jamaica are most vulnerable. These results may be viewed against the historical analysis where Jamaica, Belize and Dominican Republic are identified as the most vulnerable (see again Section 3.1). The indication then is that the emission scenario and time horizon are important factors when the comparative vulnerabilities of Caribbean countries are examined in the context of future changes in climate.

**Fig 8:**
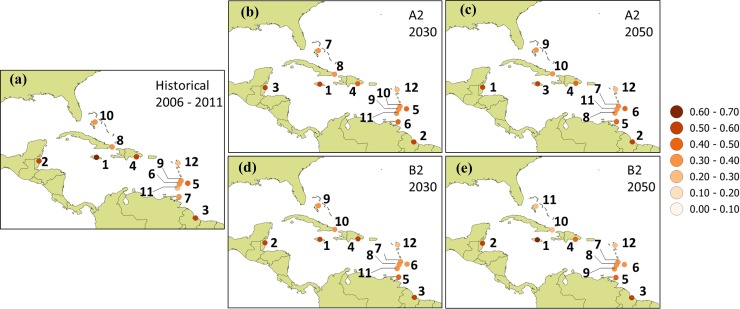
Ranking of countries based on Caribbean vulnerability scores. Equal weightings for (a) historical (2006–2011), (b)-(c) A2 scenario for 2030s and 2050s and (d)-(e) B2 scenario for 2030s and 2050s. Demographic and socioeconomic sub-indices are held constant.

In Experiment 2, the future climate sub-indices are incorporated in the CVS to analyze future comparative vulnerabilities assuming that socioeconomic and demographic factors are unchanged. Under this scenario, locations in Jamaica, Guyana and Belize are identified as most vulnerable across A2 and B2 analyses for the 2030s and 2050s but with the order of these 3 countries changing depending on time horizon and emissions scenario (see Fig 8 and S3 Appendix). Similarly, Antigua is consistently identified as the least vulnerable across all scenarios and timelines. Only Dominican Republic retains it rank (4^th^) across all scenarios and timelines. The suggestion is that the most and least vulnerable locations across the Caribbean remain so through the medium term, if changes in climate are the only consideration. Otherwise, however, relative ranking is dependent on timeline and emissions scenario being considered.

#### 3.2.5 Future Caribbean vulnerability scores: Changing climate and demographic only

Data for only 8 of 12 countries are available for analyses involving future demographic projections. Vulnerabilities defined solely on the future demographic sub-index under SSP3 (See S4 Appendix) suggests that, in order of decreasing vulnerability, the Dominican Republic, Cuba and Barbados are the most vulnerable by 2030 and 2050. In the historical analyses, Barbados and Jamaica were identified as the most vulnerable using this sub-index alone (see again Fig 4). By 2030, Barbados and Jamaica rank respectively 3^rd^ and 4^th^. Bahamas, Guyana and Belize are identified as the least vulnerable. The relative rank of all the countries remains the same between 2030 and 2050.

Table 5 shows the rank of countries for the future CVS based on changing climate under A2 and future demographic sub-index under SSP3 with constant socioeconomic sub-index (Experiment 3). Dominican Republic, Jamaica and Guyana are identified as the most vulnerable in order of decreasing vulnerability for the 2030s. For the 2050s, the Dominican Republic retains its rank as the most vulnerable followed by Belize and Jamaica. Barbados (7^th^) and the Bahamas (8^th^) are identified as the least vulnerable of the 8 locations for the 2030s and 2050s. These results show some similarity to the results from the future CVS calculated for a changing climate only (Experiment 2), in that, the locations in Jamaica and Guyana are again identified as among the most vulnerable for the 2030s and 2050s respectively. Of the 8 countries examined in Experiment 3, Cuba consistently had the lowest ranking in the future CVS analyses based on a changing climate and constant demographic and socioeconomic sub-indices (Experiment 2).

**Table 5:** Future CVS formulated using changing climate under A2, changing demographic sub-index under SSP3 and constant socioeconomic sub-index for the 2030s and 2050s.

Countries	2030s	Countries	2050s	Rank
Dominican Republic	0.538	Dominican Republic	0.503	1
Jamaica	0.518	Belize	0.476	2
Guyana	0.451	Jamaica	0.463	3
Belize	0.435	Guyana	0.420	4
Cuba	0.377	Trinidad and Tobago	0.334	5
Trinidad and Tobago	0.307	Cuba	0.329	6
Barbados	0.226	Barbados	0.219	7
Bahamas	0.224	Bahamas	0.196	8

## 4. Experiment 4

**Fig 9:**
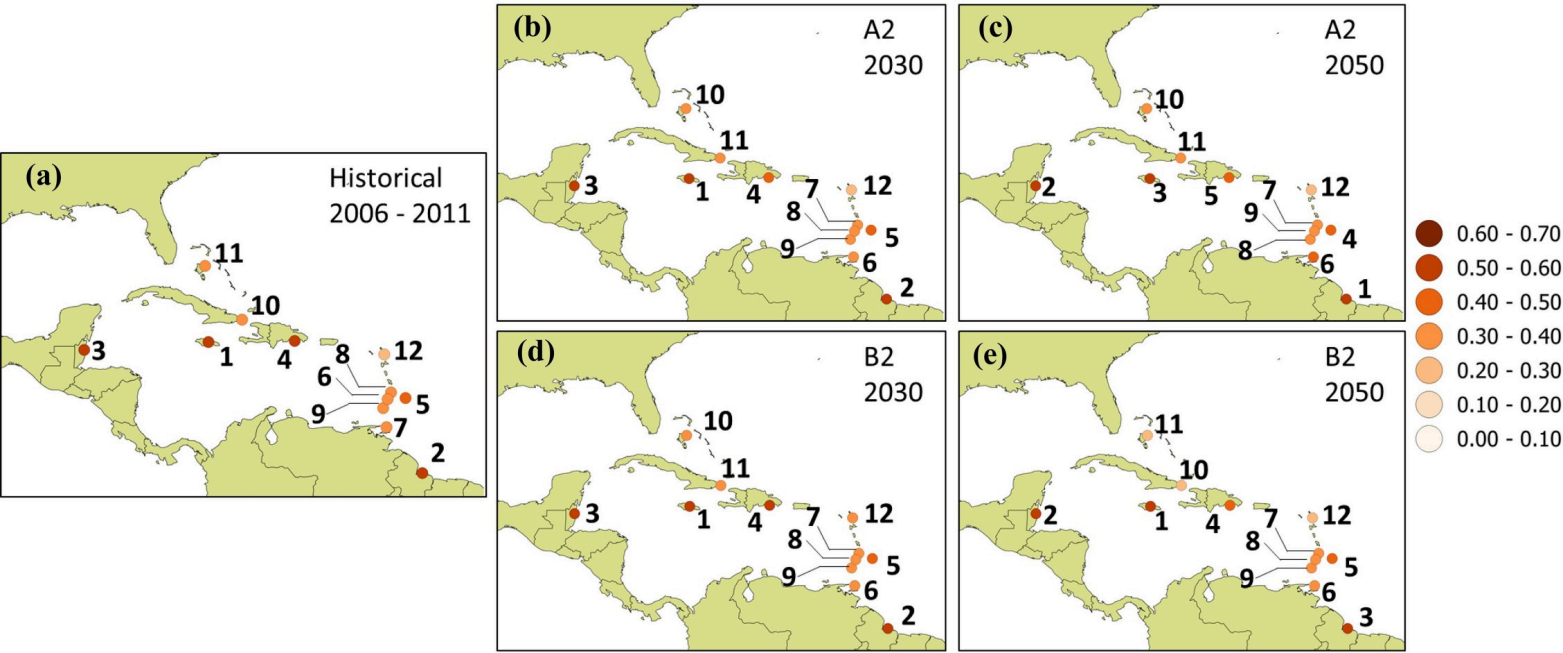
Ranking of countries based on Caribbean vulnerability scores. Weighted average for (a) historical (2006–2011), (b)-(c) A2 scenario for 2030s and 2050s relative to 2001–2015 and (d)-(e) B2 scenario for 2030s and 2050s relative to 2001–2015. Demographic and socioeconomic sub-indices are held constant.

Weightings are applied to the formulation of CVS and rankings re-calculated for the scenario of changing climate and constant demographic and socioeconomic sub-indices for the A2 and B2 scenarios. The aim is to investigate the sensitivity of the analyses to the weighting used. Fig 9 and S5 Appendix show the results of these investigations. In terms of suggested overall vulnerability, locations in Jamaica, Guyana and Belize are identified as most vulnerable by the 2030s under the A2. For the 2030s B2 analyses and for all scenarios by the 2050s, the same locations are identified as the most vulnerable but with a different order. For example, under the A2 by the 2050s Guyana and Belize are jointly the most vulnerable with Jamaica following. Under the B2 by the 2050s, the order of decreasing vulnerability is Jamaica, Belize and Guyana. It is noted that this latter order is also suggested for equal weighting, under the B2 for the 2030s and 2050s. Antigua is identified as the least vulnerable across all scenarios, formulations and timelines. Other countries in the three lowest vulnerability rankings were Cuba and Bahamas for the weighted approach across all scenarios and timelines. Recall that for equal weighting Grenada, Cuba, St. Lucia, St. Vincent and Bahamas were interchangeably among the locations identified as least vulnerable across scenarios and timeline. The suggestion is that for the most and least vulnerable, there was some insensitivity to the weightings applied in this study. However for most countries examined, scenario, index formulation and the timeline considered are all factors that can influence the rank of locations with respect to their suggested vulnerabilities.

## 5. Discussion and conclusions

This study attempts to determine *if and how past and future comparative vulnerabilities of some Caribbean countries to climate change may be altered when weather extremes are considered in the analyses*. To do so, a simple aggregate index is formulated and calculated for twelve Caribbean stations for four experiments. The four experiments capture comparative levels of vulnerabilities to climate change using combinations of historical and/or future changes in intense rain events and demographic structure, and constant socioeconomic well-being. The results suggest the following:

1. Intra-regional variations in climate matter in the determination of comparative vulnerabilities for Caribbean countries. The results of Experiment 1 suggest differences in ranking when climate is explicitly considered as a variable in determining the relative vulnerabilities. In a number of instances vulnerability increased for the CVS calculated with (versus without) a climate sub-index. For example, there were shifts in ranks for Belize (4^th^ to 2^nd^), Dominican Republic (6^th^ to 4^th^), St. Vincent (8^th^ to 6^th^), Trinidad (11^th^ to 7^th^) and Cuba (12^th^ to 8^th^). Interestingly, the particular climate extremes indicator used in this study (for heavy rainfall) appears to be a dominant influence on the suggested relative vulnerabilities for the present-day, for the CVS formulation.
2. Care must be taken when determining future vulnerabilities under a changing climate, as except for the most and least vulnerable, rankings shift dependent on scenario examined and time slice considered. This was generally true for Experiments 2 and 3 which respectively considered changing climate only (demographic and socioeconomic indices held constant), and changing climate and demographics (socioeconomic conditions held constant). The relative insensitivity of the most and least vulnerable was, however, noteworthy. For example, the locations in Jamaica and Guyana are consistently identified as among the most vulnerable for the 2030s and 2050s and in the historical analyses. This may suggest that future shifts in climate up to the medium term should be closely monitored by those already displaying extreme sensitivity and targeted actions taken to mitigate against this extreme sensitivity.
3. Changing the weightings used in the CVS formulation influenced the relative rankings of locations, but there was also some level of consistency in the countries identified as most and least vulnerable. In Experiment 4, except for Jamaica, Guyana and Belize which were identified as most vulnerable, and Antigua which emerged as least vulnerable, rankings changed dependent on scenario, timelines and weightings used.

In this study, the formulation of the CVS was guided by other aggregate indices of vulnerability found in the literature (Table 2). A deliberate attempt was, however, made to keep CVS simple in its formulation while still capturing potential variations in vulnerability to climate using climatic and non-climatic sub-indices. It was noted that of the 12 Caribbean countries examined in this study, locations in Jamaica, Guyana and Belize emerge as among the most vulnerable using historical values of the climate sub-index. This may suggest that these locations have a higher exposure to heavy rain hazards than many of the other locations examined in this study. Jamaica was again identified among the most vulnerable locations for the historical demographic sub-index suggesting an enhanced sensitivity likely due to a larger at-risk population. All three countries were also highlighted for elevated socio-economic vulnerability which likely indicates low adaptive capacity to risks posed by a changing climate. Furthermore, whether the scenario is less (more) intense rain events as generally suggested under the A2 (B2) scenario, Jamaica, Guyana and Belize are again identified as the most vulnerable locations for the 2030s and 2050s for the CVS premised on a changing climate with constant demographic and socioeconomic indices. Antigua was consistently identified as the least vulnerable.

For a comparison with another aggregate index, see again the available relative rankings for the Climate Change Vulnerability Index in Fig 1. The relative rankings for overlapping countries are not very dissimilar except for Cuba and Antigua that in this study are identified as having lower vulnerabilities and St. Vincent and Barbados that are identified here as having higher vulnerabilities. Additionally, [32] ranks Jamaica as third of 75 countries in terms of economic risk from multiple natural hazards (earthquakes, volcanoes, storms, extreme temperatures, droughts, floods, landslides, etc.). Dominican Republic (4^th^); Trinidad and Tobago (15^th^); Antigua and Barbuda (18^th^) are examples of other ranked Caribbean countries on the list [32]. [33] also lists Jamaica, Cuba, Dominican Republic and Haiti as the countries in the Caribbean with the highest disaster count in a 1950–2014 period (see their Table 2). [34] found the most disaster-prone island group to be the Greater Antilles, with Cuba, Haiti and Jamaica reporting 47, 48 and 44 disaster events respectively for 1900–1997 and 20, 20 and 9 events for 1987–1997. [34] further suggested that the Lesser Antilles have low to mid intensities of disaster.

Notably a limitation of the current study is the absence of projections of socio-economic inputs in examining future vulnerabilities. As these datasets become available at the national and subnational levels the scope for undertaking similar investigations will increase. Additionally, it is recognized that the rainfall sub-indices used in formulation of the CVS are for one station in each country (many times the airport station) as for most countries examined this was the best and/or only daily data available. There is, then, an assumption that, especially for the smaller islands, the conditions that lead to repeated days of extreme rainfall are generally felt country-wide and result from larger-scale climatic phenomenon and are not so much topographically driven. With a renewed Caribbean drive to capture more and better data on sub-daily time-scales for each territory [3], future similar work will be able to utilize a climate index with greater country-scale representation. The formulation of the CVS also includes indictors that have associated limitations and may be open to other interpretations. For example, while a country may have a relatively lower population density which this study (following other reports) suggests is indicative of a relatively lower vulnerability, a context of the actual location of this population, for example, within 2 m of the coast would suggest a higher vulnerability. There is need therefore to continue to refine this work to obtain indices that are as representative to local circumstances as possible. Additionally some of the results for the vulnerabilities suggested by individual sub-indices and the CVS are worthy of further interrogation. For example this study suggests that with respect to the socio-economic sub-index the location in Cuba is among the least vulnerable given that its longevity is the highest in the selection of countries under investigation and its gross domestic product growth rate is second highest but is offset by its lower rank (7^th^) for the gross enrolment ratio, secondary for both sexes.

In the study, the 2030s and 2050s periods overlap with the periods where respective global warming targets of 1.5° and 2.0°C above pre-industrial temperatures are expected to be attained [35]. The climate risks that Caribbean SIDS will likely face in relation to these targets are described by [35]. This study, suggests that even amidst generally high risk for the entire region, the potential impacts on individual territories will not necessarily be uniform with some SIDS having disproportionate impact. There may therefore be need to further tease out the relative vulnerabilities of different territories at these future warming levels, with the possibility to do so offered by aggregate vulnerability indices like CVS.

Finally, then, these results suggest that there is a place for the aggregate index approach in analyzing past and future comparative vulnerabilities to climate for the Caribbean. For the larger Caribbean countries the potential also exists to use these indices to differentiate vulnerabilities at the sub-national scale. The study results suggest, however, that when such indices are employed, they should account for intra-regional variations in climate. Additionally, the resulting analyses premised on aggregate indices must be accompanied by explicit explanations of the future scenario considered, the future time slice being examined, and if and how the climate information is weighted in determining the aggregate index, to aid in the interpretation of the results.

## Supporting information

S1 Appendix Correlation results between observed and modelled extreme rainfall indices, using validation period. (DOCX)

S2 Appendix Percentage change for climate indicators under the A2 scenario for the 2030s. (DOCX)

S3 Appendix CVS for the equal weighting formulation, under the A2 and B2 for the 2030s and 2050s. (DOCX)

S4 Appendix Future demographic vulnerability ranking under SSP3 for the 2030s and 2050s. (DOCX)

S5 Appendix CVS for the weighted formulation under the A2 and B2 for the 2030s and 2050s. (DOCX)
