# Supplementary material for: Caribbean climate change vulnerability: Lessons from an aggregate index approach
Source: PLoS One. 2019 Jul 10;14(7):e0219250. doi: 10.1371/journal.pone.0219250 (PMC6619692; doi:10.1371/journal.pone.0219250)
Supplement: S1 Appendix — (DOCX) [file pone.0219250.s001.docx]

**S1 Appendix.**  Correlation results between Observed and Modelled extreme rainfall indices, using validation period.

| **Stations** | **Period** | **RX1** | **RX5** | **R10** |
| --- | --- | --- | --- | --- |
| Antigua | 1984-2001 | **0.36** | **0.31** | **0.71** |
| Bahamas | 1981-2001 | 0.20 | -0.26 | **0.46** |
| Barbados | 1985-2001 | 0.25 | 0.29 | 0.29 |
| Belize | 1993-2000 | -0.09 | -0.35 | **0.38** |
| Cuba | 1991-2001 | **0.34** | **0.41** | **0.49** |
| Dominican Republic | 1986-2001 | **0.58** | **0.38** | **0.66** |
| Grenada | 1988-2001 | -0.07 | -0.20 | **0.40** |
| Guyana | 1988-2001 | 0.18 | 0.02 | 0.28 |
| Jamaica | 1987-2001 | **0.39** | **0.31** | **0.39** |
| St Lucia | 1984-2001 | 0.03 | -0.15 | -0.14 |
| St Vincent | 1994-2001 | -0.25 | 0.13 | -0.04 |
| Trinidad | 1984-2001 | -0.33 | -0.21 | 0.02 |
