# Supplementary material for: Caribbean climate change vulnerability: Lessons from an aggregate index approach
Source: PLoS One. 2019 Jul 10;14(7):e0219250. doi: 10.1371/journal.pone.0219250 (PMC6619692; doi:10.1371/journal.pone.0219250)
Supplement: S2 Appendix — (DOCX) [file pone.0219250.s002.docx]

**S2 Appendix.** Percentage change for climate indicators under the A2 scenario for the 2030s

| Countries | RX1 | Rx5 | R10 |
| --- | --- | --- | --- |
| Antigua and Barbuda | -26 | -30 | -38 |
| Bahamas | -15 | 1 | 10 |
| Barbados | -10 | -16 | -33 |
| Belize | -21 | -11 | -14 |
| Cuba | -2 | -12 | -25 |
| Dominican Republic | -36 | -25 | -35 |
| Grenada | -35 | -20 | -2 |
| Guyana | -9 | -4 | -6 |
| Jamaica | -27 | -16 | -43 |
| St. Lucia | -43 | -19 | 13 |
| St. Vincent | -47 | -20 | -19 |
| Trinidad and Tobago | 3 | -3 | -1 |
