# Supplementary material for: Caribbean climate change vulnerability: Lessons from an aggregate index approach
Source: PLoS One. 2019 Jul 10;14(7):e0219250. doi: 10.1371/journal.pone.0219250 (PMC6619692; doi:10.1371/journal.pone.0219250)
Supplement: S3 Appendix — (DOCX) [file pone.0219250.s003.docx]

**S3 Appendix.** CVS for the equal weighting formulation, under the A2 and B2 for the 2030s and 2050s.

| Country | A2-2030s | Country | B2-2030s | Country | A2 – 2050s | Country | B2 – 2050s |
| --- | --- | --- | --- | --- | --- | --- | --- |
| Jamaica | 0.594 | Jamaica | 0.585 | Belize | 0.584 | Jamaica | 0.603 |
| Guyana | 0.591 | Belize | 0.567 | Guyana | 0.569 | Belize | 0.555 |
| Belize | 0.549 | Guyana | 0.567 | Jamaica | 0.554 | Guyana | 0.520 |
| Dominican Republic | 0.492 | Dominican Republic | 0.534 | Dominican Republic | 0.451 | Dominican Republic | 0.479 |
| Barbados | 0.420 | Trinidad and Tobago | 0.406 | Trinidad and Tobago | 0.433 | Trinidad and Tobago | 0.421 |
| Trinidad and Tobago | 0.408 | Barbados | 0.379 | Barbados | 0.420 | Barbados | 0.381 |
| Bahamas | 0.352 | St. Lucia | 0.362 | St. Lucia | 0.355 | St. Lucia | 0.357 |
| Cuba | 0.348 | St. Vincent | 0.339 | Grenada | 0.340 | St. Vincent | 0.351 |
| St. Vincent | 0.347 | Bahamas | 0.328 | Bahamas | 0.335 | Grenada | 0.322 |
| St. Lucia | 0.339 | Cuba | 0.316 | Cuba | 0.332 | Cuba | 0.293 |
| Grenada | 0.316 | Grenada | 0.313 | St. Vincent | 0.332 | Bahamas | 0.274 |
| Antigua | 0.280 | Antigua | 0.281 | Antigua | 0.266 | Antigua | 0.259 |
