# Supplementary material for: Caribbean climate change vulnerability: Lessons from an aggregate index approach
Source: PLoS One. 2019 Jul 10;14(7):e0219250. doi: 10.1371/journal.pone.0219250 (PMC6619692; doi:10.1371/journal.pone.0219250)
Supplement: S4 Appendix — (DOCX) [file pone.0219250.s004.docx]

**S4 Appendix.** Future demographic vulnerability ranking under SSP3 for 2030s and 2050s

| **Countries** | **2030s** | **2050s** |
| --- | --- | --- |
| Dominican Republic | 0.632 | 0.725 |
| Cuba | 0.540 | 0.503 |
| Barbados | 0.337 | 0.334 |
| Jamaica | 0.266 | 0.291 |
| Trinidad and Tobago | 0.193 | 0.194 |
| Bahamas | 0.027 | 0.030 |
| Guyana | 0.024 | 0.020 |
| Belize | 0.016 | 0.020 |
