# Supplementary material for: Caribbean climate change vulnerability: Lessons from an aggregate index approach
Source: PLoS One. 2019 Jul 10;14(7):e0219250. doi: 10.1371/journal.pone.0219250 (PMC6619692; doi:10.1371/journal.pone.0219250)
Supplement: S5 Appendix — (DOCX) [file pone.0219250.s005.docx]

**S5 Appendix.** CVS for the weighted formulation under the A2 and B2 for the 2030s and 2050s

| Country | A2 - 2030s | Country | B2 - 2030s | Country | A2 – 2050s | Country | B2 – 2050s |
| --- | --- | --- | --- | --- | --- | --- | --- |
| Jamaica | 0.573 | Jamaica | 0.566 | Guyana | 0.545 | Jamaica | 0.579 |
| Guyana | 0.561 | Guyana | 0.543 | Belize | 0.545 | Belize | 0.522 |
| Belize | 0.518 | Belize | 0.532 | Jamaica | 0.543 | Guyana | 0.508 |
| Dominican Republic | 0.476 | Dominican Republic | 0.507 | Barbados | 0.451 | Dominican Republic | 0.466 |
| Barbados | 0.450 | Barbados | 0.420 | Dominican Republic | 0.445 | Barbados | 0.422 |
| Trinidad and Tobago | 0.390 | Trinidad and Tobago | 0.387 | Trinidad and Tobago | 0.408 | Trinidad and Tobago | 0.399 |
| St. Lucia | 0.361 | St. Lucia | 0.379 | St. Lucia | 0.373 | St. Lucia | 0.375 |
| St. Vincent | 0.357 | St. Vincent | 0.352 | Grenada | 0.365 | St. Vincent | 0.360 |
| Grenada | 0.347 | Grenada | 0.345 | St. Vincent | 0.346 | Grenada | 0.351 |
| Bahamas | 0.342 | Bahamas | 0.324 | Bahamas | 0.329 | Cuba | 0.291 |
| Cuba | 0.332 | Cuba | 0.308 | Cuba | 0.320 | Bahamas | 0.284 |
| Antigua | 0.299 | Antigua | 0.300 | Antigua | 0.289 | Antigua | 0.283 |
